# WHAM!: a web-based visualization suite for user-defined analysis of metagenomic shotgun sequencing data

**DOI:** 10.1186/s12864-018-4870-z

**Published:** 2018-06-25

**Authors:** Joseph C. Devlin, Thomas Battaglia, Martin J. Blaser, Kelly V. Ruggles

**Affiliations:** 1 Sackler Institute of Graduate Biomedical Sciences, New York School of Medicine, New York, NY USA; 2 Division of Translational Medicine, Department of Medicine, New York School of Medicine, New York, NY USA; 3 Department of Microbiology, New York School of Medicine, New York, NY USA; 4 Applied Bioinformatics Laboratories, New York School of Medicine, New York, NY USA

**Keywords:** Microbiome, Metatranscriptomic, Data exploration, RShiny, DNA analysis, Expression analysis

## Abstract

**Background:**

Exploration of large data sets, such as shotgun metagenomic sequence or expression data, by biomedical experts and medical professionals remains as a major bottleneck in the scientific discovery process. Although tools for this purpose exist for 16S ribosomal RNA sequencing analysis, there is a growing but still insufficient number of user-friendly interactive visualization workflows for easy data exploration and figure generation. The development of such platforms for this purpose is necessary to accelerate and streamline microbiome laboratory research.

**Results:**

We developed the Workflow Hub for Automated Metagenomic Exploration (WHAM!) as a web-based interactive tool capable of user-directed data visualization and statistical analysis of annotated shotgun metagenomic and metatranscriptomic data sets. WHAM! includes exploratory and hypothesis-based gene and taxa search modules for visualizing differences in microbial taxa and gene family expression across experimental groups, and for creating publication quality figures without the need for command line interface or in-house bioinformatics.

**Conclusions:**

WHAM! is an interactive and customizable tool for downstream metagenomic and metatranscriptomic analysis providing a user-friendly interface allowing for easy data exploration by microbiome and ecological experts to facilitate discovery in multi-dimensional and large-scale data sets.

**Electronic supplementary material:**

The online version of this article (10.1186/s12864-018-4870-z) contains supplementary material, which is available to authorized users.

## Background

As metagenomic and metatranscriptomic shotgun sequencing data become both less expensive to generate and more readily available, researchers have turned to automated pipelines such as MetaPhlAn [1], HUMAnN2 [2, 3] MEGAN [4] and SAMSA [5] for annotation and analysis. While these applications provide high quality functional and taxonomic annotations, a computational hurdle still exists between the data output and biologically interpretable results. Output formats from annotation pipelines are typically cumbersome tables and large matrices of genes, assigned taxa, and abundance or expression levels. Researchers then must sift through the data for their genes of interest to test their stated hypotheses. Further because of the size and density of information, exploration of the data presents an even more overwhelming task for experimentalists, inhibiting data-driven discovery.

Concurrently with the increasing interest in the field, many of the tools described above have been employed to analyze and characterize the human microbiome. Two widely used tools, HUMANn2 and QIIME2, provide extensive frameworks for gene annotation and taxonomic analysis, respectively. However, both of these tools have limitations for downstream visualization and user-based data exploration. While HUMAnN2 includes a visualization script to generate relative abundance plots for a particular pathway or gene family of interest, users are limited in figure customization and must use the command line. Requiring users to specify the feature of interest hinders exploration of the data set in its entirety. However, other platforms such as QIIME2 have recognized the utility of command line independence and user-defined exploration of sequencing data. A novel feature of the QIIME2 platform includes a Graphical User Interface (GUI)-based Shiny derivative where users can visualize taxonomic information and download high-quality figures. Nevertheless, QIIME users are limited to taxonomic investigations and therefore miss the opportunity to correlate gene expression observations with taxonomic abundance. In addition to these commonly used resources, new tools and methods are continuously being developed to deal with the challenges of visualizing these complex datasets. Several R-packages or command line tools exist for this purpose, including MG-RAST [6], CAMERA [7], and ASAR [8]. Others only focus only on 16S rRNA sequence data input and are unable to accommodate shotgun metagenomics data containing information on both taxa and functional elements [9–13]. Therefore, there is a growing a need for tools addressing the specific challenges biomedical experts face when analyzing metagenomics data.

Our Workflow Hub for Automated Metagenomic Exploration (WHAM!) aims to provide a platform for simple and intuitive exploration and targeted analysis of metagenomic sequencing data. Our platform requires no computational background or processing on the part of the user to generate publication-quality figures. Furthermore, this application allows users to interactively explore their dataset for patterns and changes in expression or taxonomic composition while also providing a platform for analyzing specific biological features and their taxonomic contributors.

## Implementation

### WHAM! UI architecture

WHAM! is described here as an easy to use, web-based, R-shiny application that generates publication-quality figures for metagenomic sequencing analyses (https://ruggleslab.shinyapps.io/wham_v1/). The application employs a number of R packages including, ggplot2 [14], psych [15], gplots [16], and plotly [17] for visualization (For source code and full list of packages and dependencies please see https://github.com/ruggleslab/jukebox/tree/master/wham_v1). However, all dependencies are packaged within the application, so users only need web access and input data. Currently, the application accepts two input options, based on commonly used metagenomics pipelines and the platform is open to adding additional input options as they are developed by the community. The first is a tab-delimited output of gene families, pathways or Gene Ontology (GO) terms and their abundance or expression levels in the specified format shown in Additional file 1: Table S1. This format is based on the Huttenhower Biobakery pipeline [18] which is comprised of a suite of tools including FastQC, Kneaddata [19], MetaPhlAn [1] and HUMAnN2 [2, 3]. We chose this pipeline, in part, because the next iteration of the human microbiome project uses a workflow that includes Biobakery-based tools [20] and a curated database of metagenomics studies which have been processes through this pipeline are available through the Bioconductor ExperimentHub platform [21]. Creating user-friendly web-tools downstream of these analyses steps will allow researchers to explore the ongoing large-scale metagenomics projects without having to do the computational heavy lifting. The second input option is the European Bioinformatics Institute (EBI) Metagenomics service, in which the user can upload up to two files containing functional features (Interpro protein families, GO terms, etc.) and/or a taxa file, in the specified formats shown in Additional file 2: Table S2.

**Fig. 1:**
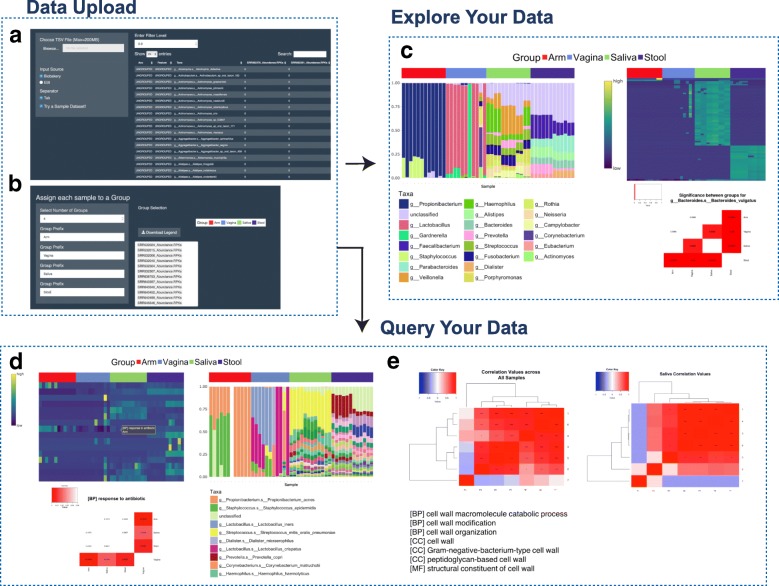
WHAM! Analysis Modules. **a** Metagenomic functional and taxonomic relative abundance matrices are uploaded and a subset of the data can be visually inspected. **b** User assignment of samples to experimental groups for downstream analysis. **c** The “Explore Your Data” module allows for interactive taxa and feature-level exploration and differential expression across user-defined groups. **d**, **e** Representation of the “Query Your Data” module that aids in hypothesis-driven exploration and visualization, in which differential expression, taxonomic composition, and correlation analysis are carried out on user-specified feature lists

Once uploaded, the file(s) are automatically previewed showing 25 searchable rows of data on the main application page to allow for visual inspection (Fig. 1a). A variance filter slider control is provided for filtering out low variance features in order to speed up differential abundance calculations and visualizations based on a variance percentile cutoff. Users can then navigate to the ‘Groups’ tab where they are prompted to manually separate their samples into as many as 10 experimental groups allowing for automated statistical comparisons between experimental groups in the downstream analysis (Fig. 1b).

### Pipeline architecture and visualization methods

Methods for the analysis of metagenomics data are rapidly being developed to meet the need of the community (see reviews [22–24]). The choice of statistical methods, in particular, must be tailored to the specific challenges inherent to metagenomics data analysis. For differential expression analysis, we chose the ANOVA-Like Differential Expression (ALDEx2) method, which takes into account within-condition variation, the compositional characteristics of high-throughput sequencing data and multiple testing corrections. This method evaluates differential expression between experimental groups using a combination of statistical significance and effect size estimates, both of which are included in our pipeline [22]. The WHAM! ‘Explore Your Data’ module has user input sliders for absolute effect size selection and Wilcoxon test *p*-value cutoffs to isolate meaningful findings in the data. A non-parametric Spearman correlation analysis was chosen for our cross correlation tests, with Benjamini-Hochberg correction for false discovery rates (FDR) [25].

Further, we have carefully considered the options available for metagenomic data visualization during application development. In terms of visualization, we have chosen to focus on a combination of stacked bar plots (for taxa contribution) and heatmaps (for relative abundance, correlation analysis and pairwise statistics). Stacked bar plots are able to efficiently represent the proportion of taxa present in each sample across many metagenomes and are commonly used in microbiome studies. Heatmaps are particularly useful in highlighting the taxa and gene abundance in a collection of samples or for taxa correlation plots, where other methods such as box or bar plots can become cumbersome [23].

### Data exploration

**Fig. 2:**
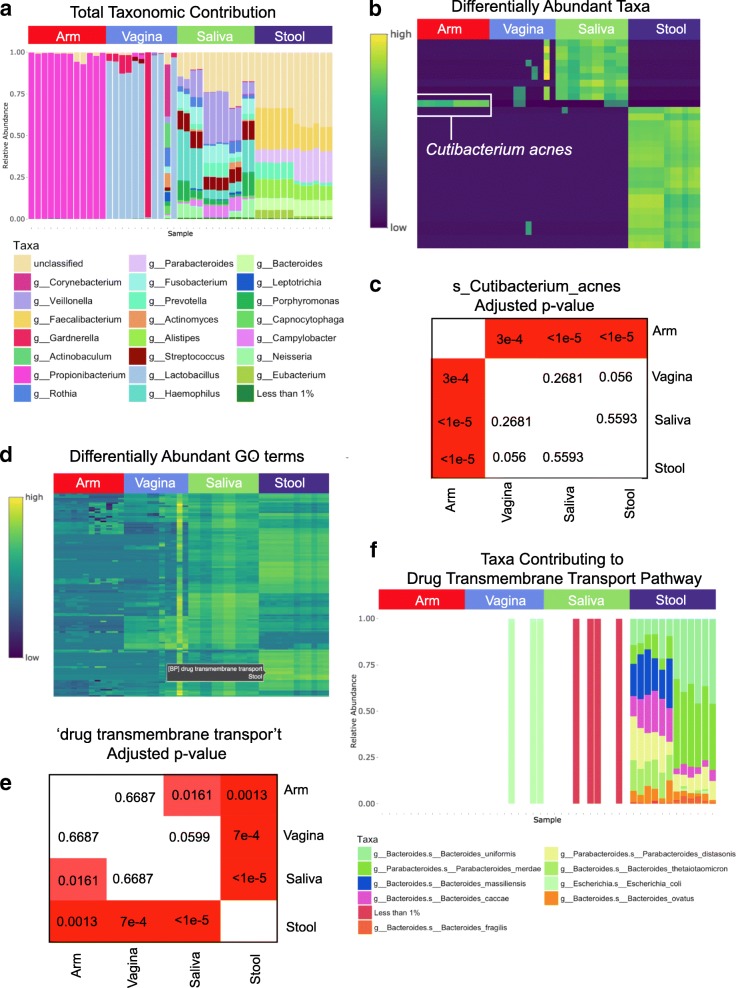
Exploratory modules show broad differences in taxa and GO-terms across body sites. Relative abundance levels of all genera detected in 47 human microbiome samples across four body sites: arm, saliva, stool, and vagina. **a** Sample plot downloaded from the ‘Explore Taxa’ module including a full list of the relevant genera displayed and their relative abundance levels across the user-specified groups. **b**, **c** Interactive sample heatmap showing differentially abundant taxa across body sites with a plot of pairwise adjusted *p*-values for *Cutibacterium acnes.* **d** Sample plot downloaded from the ‘Explore Features’ module showing the differentially abundant GO terms across groups. **e** Pairwise adjusted p-values for the drug transmembrane transport GO term, obtained by selecting the heatmap feature in **d**. **f** Taxa contributing to the drug transmembrane transport GO term

WHAM! has several built-in calculation modules for both data exploration and hypothesis-driven analyses (Fig. 1). Within the “Explore Your Data” module, users can navigate to the subtabs ‘Explore Taxa’ and ‘Explore Features’, which provide users with a global view of the functional and taxonomic composition of their dataset by visualizing all gene families, pathways or GO term-based classification and taxa present according to their relative abundance (Fig. 1c, Fig. 2). Users can analyze their taxa at different levels (e.g. genus, species, class) and a differential abundance analysis is also automatically completed to identify taxa that significantly differ across groups, using the ALDEx2 differential expression test [22]. Features found to be significantly different across any group comparison based on user supplied adjusted *p*-value and effect size cutoffs are then visualized as a heatmap (Fig. 2b). Hovering over and clicking on a specific feature in the heatmap expands the results below to show the pairwise adjusted p-value significance across groups (Fig. 1c, Fig. 2c). The ‘Explore Features’ tab also completes the differential abundance analysis again using the ALDEx2 R package, creating an associated heatmap for significantly changing genes, pathways or GO terms based on user-defined adjusted p-value and effect size cutoffs (Fig. 2d). Clicking on a feature in the heatmap visualizes the statistical differences across groups (Fig. 2e) and a breakdown of taxa contributing to the feature abundance as stacked bar plots to better understand which microbial taxa are contributing to the differentially abundant features in each sample (Fig. 2f). Both the heatmap and stacked bars are interactive, where hovering over any of the plot elements displays the corresponding gene and/or taxa details.

### Hypothesis-driven analysis

**Fig. 3:**
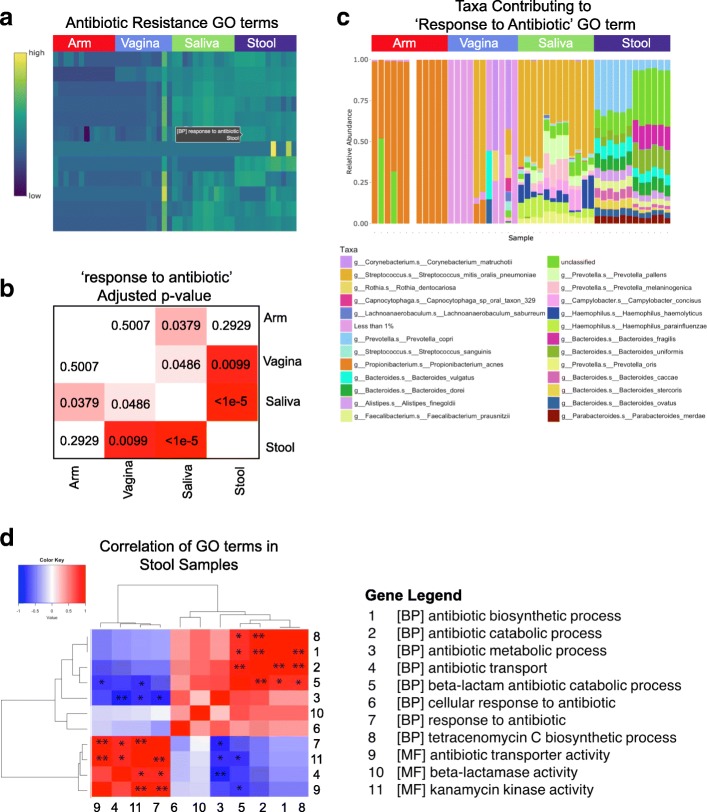
Query modules allow for in-depth exploration of antibiotic resistance. Hypothesis driven visualization based on a user-defined list of antibiotic resistance-related families in the 47 human microbiome samples indicated in Fig. 2. **a** sample output from the ‘Query Your Data’ module, including relative abundance levels of antibiotic resistance related GO-terms across samples. **b** pairwise statistical significance between sites for selected GO terms. **c** Taxa contributing to specific GO terms across samples. **d** Correlation of pathways in stool samples for a user-defined gene list aimed to elucidate relationships in abundance profiles across antibiotic-resistance mechanisms. Asterisks indicate statistical significance (*p < 0.05, **p < 0.01, ***p < 0.001)**.** Columns/rows are labeled as shown to the right

The ‘Query Your Data’ module then provides an infrastructure for user-friendly and interactive hypothesis-driven analysis (Fig. 1d). In the ‘Feature Search’ tab, users are prompted to select features of interest which automatically generates an interactive heatmap plotting the relative abundance levels for each selected gene or pathway across samples (Fig. 3a). Clicking on a feature in the heatmap propagates a stacked bar plot showing taxa abundance contributing to that gene or GO term (Fig. 3c) and, in the case where groups significantly differ in the abundance of that feature and an adjusted *p*-value matrix showing the pairwise comparisons (Fig. 3b). Lastly, the ‘Correlation’ tab calculates the pairwise correlations between user-defined genes across all samples and within each experimental group to provide insight into the relative relationships between biological features across all samples and under different experimental conditions (Fig. 3d). Correlation values and their significance are calculated using Spearman correlation and plotted as a clustered heatmap (using hclust algorithm defaults [26]) with significance levels indicated by an asterisk. This module allows, not only for a user-driven exploration of specific features of interest in the dataset, but also for the creation of publication quality figures and statistics. Due to the nature of EBI-related input, taxa contribution calculations specific to functional features are not available for both modules.

## Results

To demonstrate the utility of WHAM!, we used two independent, publicly available test datasets. The first was derived from 47 human microbiome samples from four body sites made available by the Human Microbiome Project (HMP) [27]. Shotgun metagenomic sequencing data were processed through an analysis pipeline utilizing the Huttenhower Biobakery pipeline [18], including FastQC, Kneaddata [19], MetaPhlAn [1] and HUMAnN2 [2, 3] to obtain an annotated gene abundance matrix. After host decontamination and quality filtering, the estimated counts in each sample were calculated by multiplying the relative abundances for each feature by the total sum of profiled counts. Following count estimation, the gene family identifiers were further collapsed by GO term mapping via the “humann2_regroup_table” function provided within HUMANn2. This dataset has been mounted as a test case to our web-app in the ‘Try a Sample Dataset’ mode on the application homepage. Although an already well-studied dataset, our analysis of these HMP sequencing data highlights the utility and exploratory capabilities provided by our visualization suite. As expected, body sites vary widely in the taxonomic species present and in the abundance of these taxa (Fig. 2a). Arm samples were dominated by the genus *Cutibacterium* (previously classified as *Propionibacterium)*, which was also observed in the original HMP analysis (Fig. 2b, c) [27]. Furthermore, stool and saliva samples exhibited much greater microbial diversity when compared to arm and vaginal samples, at the depth of resolution provided in the original data (Fig. 2a). As demonstrated, WHAM! is able to readily identify and visualize taxonomic differences based on group classifications which could include varied diets, drug treatment groups, disease states, or any other user-defined classification. We can similarly explore the GO term abundance across samples using the ‘Explore Features’ tab, automatically identifying differentially abundant GO terms across samples based on user-controlled *p*-value and effect size cutoffs (Fig. 2d). Of those found to be significantly different, several antibiotic resistance-related GO terms were represented, including drug transmembrane transport, differing between stool and all other body sites tested (Fig. 2e). The taxa contributing to the abundance of this pathway also differed between sites, with high diversity, including *E. coli and Bacteroides,* found in stool samples (Fig. 2f).

Because of our interest in the emergence of antibiotic resistance, we chose to explore our test data set for patterns in pathway abundances for antibiotic resistance mechanisms based on GO-term categories. By searching for these keywords in the ‘Feature Search’ tab, we detected several antibiotic resistance-related GO-term categories across the four body sites (Fig. 3a). Clicking on the features in the heatmap revealed significant differences in relative abundance levels of a subset of GO terms across the four body sites. These included the ‘response to antibiotic’ GO-term, which was significantly different in abundance in comparisons between stool and vagina, stool and saliva, vagina and saliva, and arm and saliva (Fig. 3b). Our analysis also demonstrates relatively high abundance levels of antibiotic resistance gene families in saliva and a wide dispersion of these gene families in stool samples (Fig. 3a).

Further investigation via the ‘Feature Search’ tab also provided taxonomic identification corresponding to the differences in ‘response to antibiotic’ GO-term abundance across the four body sites. In arm samples, the ‘response to antibiotic’ GO-term was almost exclusively present in *C. acnes*, while in saliva and stool samples the contributing taxa were more diverse, with the highest prevalence occurring in *Streptococcus oralis* in saliva and *Prevotella copri* in stool (Fig. 3c). Such observations in other data sets can address a number of biologically relevant questions, including how commensal bacteria contribute to the spread of antibiotic resistance, and how particular bacterial species are able to inhabit multiple different body sites, and whether or not their attributes differ across body sites.

Correlation analyses of functional features can enable users to obtain information about shared selection, or interactions between gene families, according to abundance patterns across different classification groups in the studied datasets. From this information, the highly correlated antibiotic transporter activity (GO term 9), kanamycin kinase activity (GO term 11), and response to antibiotic (GO term 7) pathways, suggest shared selection. These three pathways also were found to be anti-correlated with antibiotic metabolism (GO term 3) and beta-lactam antibiotic catabolism (GO term 5) (Fig. 3d). Establishing and evaluating these relationships in real time provides the opportunity to test and generate on-the-fly hypotheses by biomedical experts.

**Fig. 4:**
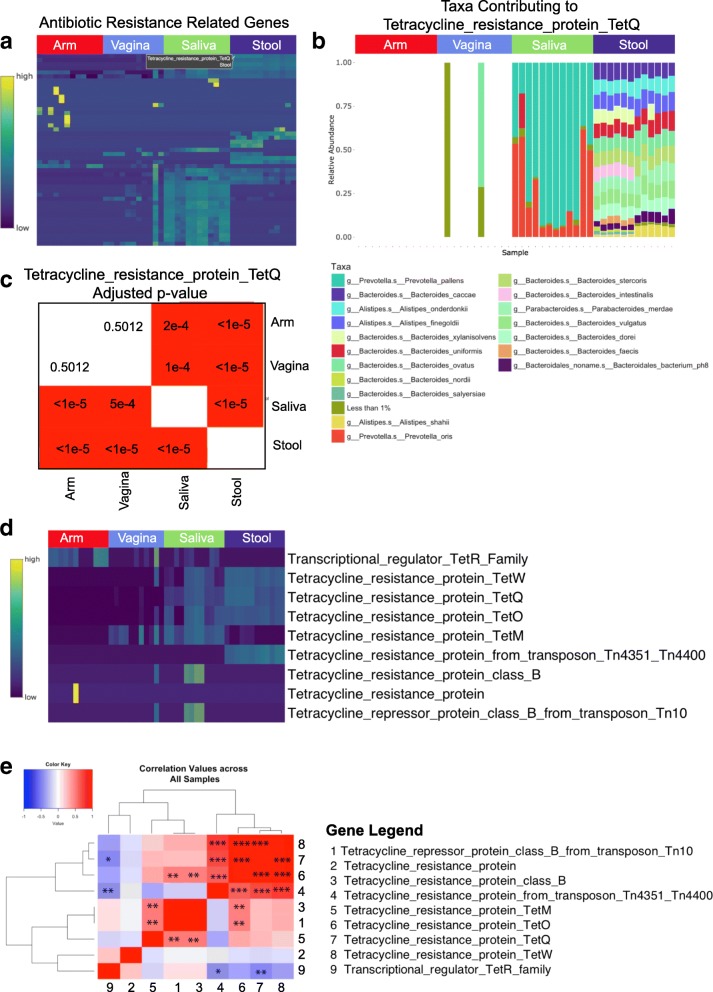
Gene family level analysis of antibiotic resistance across body sites. **a** Relative abundance of 114 user-selected Uniref90 gene family identifiers mapping to the “Response to Antibiotic” GO term. **b** Associated taxonomic contribution indicates greater diversity in stool samples compared to saliva samples. **c** Statistical details for the selected Tetracycline resistance protein TetQ gene family across 47 human microbiome samples from four body sites. Differential abundance (**d**) and cross correlation (**e**) analysis of tetracycline resistance gene families indicate prevalence of resistance mechanisms in both body sites. Columns/rows are labeled as shown to the right. Asterisks indicate statistical significance (*p < 0.05, **p < 0.01, ***p < 0.001)

Based on our findings at the GO-term level, we then investigated these samples at the gene family level, further demonstrating the utility of our tool at analyzing specific gene features in addition to a broad-level feature analysis. Analysis of 114 Uniref90 gene families that mapped to the ‘response to antibiotic’ GO-term based on the HUMANn2 mapping files showed relatively high levels of antibiotic resistance gene families in saliva and stool, with scattered extreme values also found in arm samples (Fig. 4a). Targeting a specific gene, the Tetracycline resistance protein TetQ, we found that the contributions in saliva came primarily from *Prevotella pallens* with more diverse contributions found in stool samples (Fig. 4b). There were significant differences in abundance levels occurring in all pairwise body site comparisons with the exception of the comparison between arm and vagina (Fig. 4c). Focusing further on the tetracycline resistance genes, there was shared expression in stool and saliva samples with non-zero abundance of tetracycline resistance protein class B found in saliva only (Fig. 4d). Cross comparison of the tetracycline gene families identified high correlation for a subset of genes (TetQ, TetW, TetO) (Fig. 4e), all found to be abundant across stool samples.

**Fig. 5:**
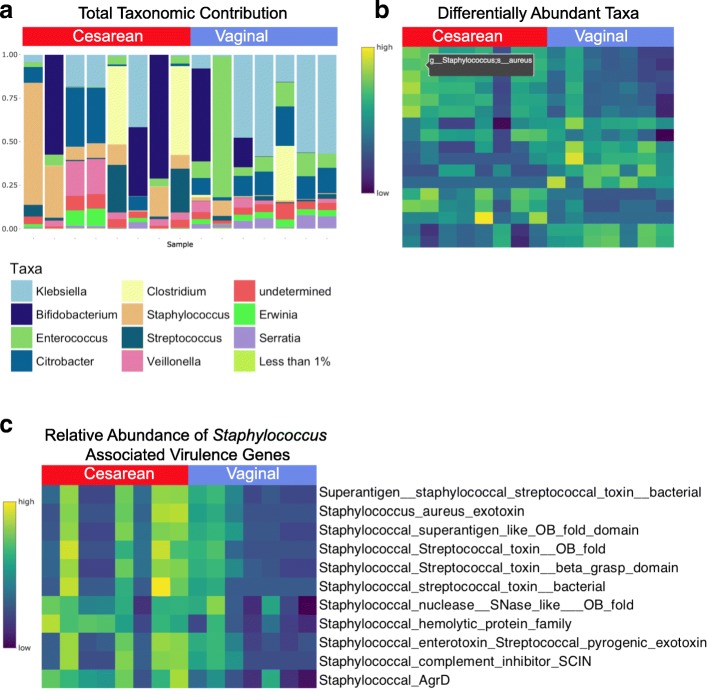
Antibiotic resistance in a study of premature babies (Rose et al., 2017). **a** Taxonomic contributions across cesarean and vaginal delivery samples propose clear differences in microbial genera. **b** Statistically significant differentially abundant taxa are visualized as a heatmap, highlighting a clinically relevant strain of *Staphylococcus aureus*. **c** Differentially abundant *Staphylococcus*-associated virulence genes demonstrate clear differences in early life microbial environment in preterm infants based on delivery mode

Lastly, we demonstrate the use of WHAM! for exploration and visualization of a second test dataset derived from the EBI metagenomic service describing the metagenomic profiling of 15 preterm infants [28]. We used the ‘Explore Your Data’ module to visualize relevant taxa present and the relative abundance of taxa in the babies born via vaginal or cesarean delivery (Fig. 5a). This analysis identified 17 taxa that differed significantly between experimental groups, including clinically important strains of Staphylococcus, such as *S. aureus*, which was significantly more abundant with cesarean delivery (adjusted *p* = 0.005) (Fig. 5b). Further analysis using the ‘Explore Features’ tab identified several Staphylococcus associated virulence proteins including a Staphylococcal hemolytic protein family and Staphylococcal AgrD which is involved in quorum-sensing signaling to release exoproteins involved in virulence [29] (Fig. 5c**)**. Both features were identified as differentially abundant between the conditions (adjusted *p* = 0.0014 and *p* = 0.0042 respectively). We provide this information to illustrate how WHAM! can facilitate the discovery of taxa and their genes that could be of clinical significance. Although *S. aureus* can be an important pathogen in infants [30] the available metadata do not permit assessment of its clinical significance in this study.

These implementation examples demonstrate how WHAM! can be applied to metagenomics data to easily identify and visualize biologically relevant relationships and to generate novel hypotheses. Recently developed tools, Metaviz [31], BURRITO [32] and MetaComp [33], address similar challenges, however, WHAM! has several important differences. Although visually striking and useful, Metaviz focuses on taxonomic analysis without factoring in biological processes, gene features or pathways [31]. Like WHAM!, BURRITO enables uses to interactively explore their metagenomics data, but lacks the capability of feature searching and hypothesis testing and provides fewer statistical tests for relative abundance across groups when compared with WHAM! [32]. MetaComp has robust statistics and accepts a range of inputs, but it requires an external download and installation, which can lead to unexpected issues depending on the user’s compute platform [33]. WHAM! allows for web-based hypothesis generation based on both taxa and functional features, permitting on-the-fly confirmation and figure generation, substantially adding to the current suite of tools available for metagenomic analysis.

## Conclusions

WHAM! is an interactive and customizable tool for data exploration, hypothesis generation and figure generation for downstream metagenomics and metatranscriptomics analysis. Offering these capabilities as an R Shiny web tool provides a user-friendly interface allowing for easy data exploration by ecologists and microbiologists to streamline discovery in multi-dimensional and large-scale data sets. Overall, WHAM! strives to provide users with the opportunity for in-depth exploration and targeted analysis of metagenomic and metatrascriptomic sequencing information with special emphasis on microbiome-related investigations. As demonstrated, the ease and utility of the WHAM! visualization suite enables users to explore patterns in the microbiome, to understand relationships between taxonomic communities and the processes in which they engage. For 16S rRNA taxonomic analysis, QIIME and Mothur have dominated the field as user friendly comprehensive bioinformatics pipelines for microbial taxonomic analysis [34, 35]. QIIME2 improved upon the pipeline, not only in the taxonomic inference algorithm [36], but also in its user interface, now including interactive web-based visualization and no longer requiring the use of a command line interface [37]. Currently, there is a growing, but insufficient number of tools that allow for real-time exploratory visualization of complex shotgun metagenomics data that are designed specifically for biomedical scientists and medical professionals lacking computational training. WHAM! helps to fill this gap and we will continue to expand upon the capabilities of our tool by increasing the allowable input data structures and supported statistical packages to reflect the evolving analysis methods as they are adopted by the field.

## Availability and requirements

Project name: Workflow Hub for Automated Metagenomic Exploration (WHAM!)

Project home page: https://ruggleslab.shinyapps.io/wham_v1/

Operating system: Platform independent.

Programming Language: R/Rshiny.

Other requirements: None.

License: None.

Any restrictions to use by non-academics: None.

## Additional files

Additional file 1: **Table S1.** Huttenhower Biobakery formatted sample input data input. Data derived from reanalysis of a subset of HMP samples [27]. (PDF 133 kb)

Additional file 2: **Table S2.** EBI Metagenomics formatted sample input data input. **a** Taxonomic and **b** GO-term level data derived from Rose et al. [28]. (PDF 115 kb)
