# Supplementary material for: WHAM!: a web-based visualization suite for user-defined analysis of metagenomic shotgun sequencing data
Source: BMC Genomics. 2018 Jun 25;19:493. doi: 10.1186/s12864-018-4870-z (PMC6019711; doi:10.1186/s12864-018-4870-z)
Supplement: Supplementary file 1 — Table S1. Huttenhower Biobakery formatted sample input data input. Data derived from reanalysis of a subset of HMP samples [27]. (PDF 133 kb) [file 12864_2018_4870_MOESM1_ESM.pdf]

Table S1. Huttenhower Biobakery formatted sample input data input.

Acc	Feature	Taxa	Sample 1	Sample 2	Sample 3	Sample 4	Sample 5	Sample 6	Sample 7	Sample 8
UNGROUPED	UNGROUPED	g__Abiotrophia.s__Abiotrophia_defectiva	0	0	0	0	332	348	183	281
UNGROUPED	UNGROUPED	g__Actinobaculum.s__Actinobaculum_sp_oral_taxon_183	0	0	0	0	0	0	0	0
UNGROUPED	UNGROUPED	g__Actinomyces.s__Actinomyces_graevenitzii	0	0	0	0	6582	5703	833	2416
UNGROUPED	UNGROUPED	g__Actinomyces.s__Actinomyces_johnsonii	0	0	0	0	1287	828	0	0
2001295	[BP] malonyl-CoA biosynthetic process	g__Streptococcus.s__Streptococcus_intermedius	0	0	0	0	0	2	0	0
2001295	[BP] malonyl-CoA biosynthetic process	g__Streptococcus.s__Streptococcus_mitis_oralis_pneumoniae	0	0	0	0	202	230	74	329
2001295	[BP] malonyl-CoA biosynthetic process	g__Streptococcus.s__Streptococcus_parasanguinis	0	0	0	0	68	56	11	111
2001295	[BP] malonyl-CoA biosynthetic process	g__Streptococcus.s__Streptococcus_peroris	0	0	0	0	2	3	0	24
2001295	[BP] malonyl-CoA biosynthetic process	g__Streptococcus.s__Streptococcus_pseudopneumoniae	0	0	0	0	0	0	0	0
2001295	[BP] malonyl-CoA biosynthetic process	g__Streptococcus.s__Streptococcus_salivarius	0	0	0	0	67	66	2	44
2001295	[BP] malonyl-CoA biosynthetic process	g__Streptococcus.s__Streptococcus_sanguinis	0	0	0	0	11	11	0	2
2001295	[BP] malonyl-CoA biosynthetic process	g__Streptococcus.s__Streptococcus_sp_SK140	0	0	0	0	0	0	0	56
2001295	[BP] malonyl-CoA biosynthetic process	g__Streptococcus.s__Streptococcus_thermophilus	0	0	0	0	2	5	0	0
2001295	[BP] malonyl-CoA biosynthetic process	g__Streptococcus.s__Streptococcus_tigurinus	0	0	0	0	4	5	0	16
2001295	[BP] malonyl-CoA biosynthetic process	g__Streptococcus.s__Streptococcus_vestibularis	0	0	0	0	0	7	0	0
2001295	[BP] malonyl-CoA biosynthetic process	g__Sutterella.s__Sutterella_wadsworthensis	0	0	0	0	0	0	0	0
2001295	[BP] malonyl-CoA biosynthetic process	g__Treponema.s__Treponema_denticola	0	0	0	0	0	0	0	0
2001295	[BP] malonyl-CoA biosynthetic process	g__Treponema.s__Treponema_lecithinolyticum	0	0	0	0	0	0	0	0
2001295	[BP] malonyl-CoA biosynthetic process	g__Treponema.s__Treponema_medium	0	0	0	0	0	0	0	0
2001295	[BP] malonyl-CoA biosynthetic process	unclassified	0	0	0	0	60	63	0	120
