# Supplementary material for: WHAM!: a web-based visualization suite for user-defined analysis of metagenomic shotgun sequencing data
Source: BMC Genomics. 2018 Jun 25;19:493. doi: 10.1186/s12864-018-4870-z (PMC6019711; doi:10.1186/s12864-018-4870-z)
Supplement: Supplementary file 2 — Table S2. EBI Metagenomics formatted sample input data input. a Taxonomic and b GO-term level data derived from Rose et al. [28]. (PDF 115 kb) [file 12864_2018_4870_MOESM2_ESM.pdf]

Table S2. EBI Metagenomics formatted sample input data input

a

Acc	Feature	Sample 1	Sample 2	Sample 3	Sample 4	Sample 5	Sample 6	Sample 7	Sample 8
IPR000011	Ubiquitin/SUMO-activating enzyme E1	0	0	0	0	0	0	0	0
IPR000014	PAS domain	699	289	668	137	1364	1254	418	518
IPR000015	Outer membrane usher protein	11075	13314	1675	8	34255	33647	15053	16385
IPR000021	Hok/gef cell toxic protein	630	457	82	0	522	505	509	592
IPR000022	Carboxyl transferase	140	2186	740	3295	556	494	916	593
IPR000023	Phosphofructokinase domain	1683	954	2041	955	1760	1721	1484	2280
IPR000026	Guanine-specific ribonuclease N1/T1/U2	15	2	98	14	0	0	2	15
IPR000031	PurE domain	1426	1189	1832	1690	1240	1244	829	861
IPR000032	Phosphocarrier protein HPr-like	1511	1988	1164	1007	1650	1685	1549	2244
IPR000035	Alkylbase DNA glycosidase, conserved site	47	46	0	0	146	111	97	117
IPR000036	Peptidase A26, omptin	470	0	0	0	152	100	103	15
IPR000037	SsrA-binding protein	513	657	735	843	542	511	477	505
IPR000043	Adenosylhomocysteinase	2	62	46	20	107	102	76	54
IPR000045	Prepilin type IV endopeptidase, peptidase domain	395	356	225	62	888	898	709	914
IPR000049	Electron transfer flavoprotein, beta-subunit, conserved site	79	8	0	1	11	13	17	0
IPR000055	Restriction endonuclease, type I, HsdS	641	583	609	1174	802	860	309	318
IPR000056	Ribulose-phosphate 3-epimerase-like	1804	2762	3003	2831	1580	1513	2655	2696
IPR000058	Zinc finger, AN1-type	0	2	0	0	0	0	6	0
IPR000059	NUDIX hydrolase, NudL, conserved site	64	30	40	4	145	133	91	117
IPR000060	BCCT transporter family	8085	1115	2664	3735	9349	9267	2328	3560

Supplementary Table 2. EBI Metagenomics formatted sample input data input

b

Taxa	Sample 1	Sample 2	Sample 3	Sample 4	Sample 5	Sample 6	Sample 7
Root	1251	1832	1238	2255	1760	1693	1595
Root;k__Bacteria;p__Acidobacteria;c__Acidobacteria-6;o__iii1-15;f__;g__;s__	0	0	1	0	0	0	0
Root;k__Bacteria;p__Actinobacteria;c__Acidimicrobiia;o__Acidimicrobiales;f__C111;g__;s__	0	0	1	0	0	0	0
Root;k__Bacteria;p__Actinobacteria;c__Actinobacteria;o__Actinomycetales;f__;g__;s__	0	9	4	8	0	0	2
Root;k__Bacteria;p__Actinobacteria;c__Actinobacteria;o__Actinomycetales;f__ACK-M1;g__;s__	0	0	0	1	0	0	0
Root;k__Bacteria;p__Actinobacteria;c__Actinobacteria;o__Actinomycetales;f__Actinomycetaceae;g__Actinomyces;s__	0	13	0	17	0	0	0
Root;k__Bacteria;p__Actinobacteria;c__Actinobacteria;o__Actinomycetales;f__Actinomycetaceae;g__Arcanobacterium;s__	0	0	1	0	0	0	0
Root;k__Bacteria;p__Actinobacteria;c__Actinobacteria;o__Actinomycetales;f__Actinomycetaceae;g__Varibaculum;s__	0	0	0	2	0	0	5
Root;k__Bacteria;p__Actinobacteria;c__Actinobacteria;o__Actinomycetales;f__Actinospicaceae;g__;s__	0	0	2	0	0	0	0
Root;k__Bacteria;p__Actinobacteria;c__Actinobacteria;o__Actinomycetales;f__Brevibacteriaceae;g__Brevibacterium;s__	0	0	0	0	0	0	0
Root;k__Bacteria;p__Actinobacteria;c__Actinobacteria;o__Actinomycetales;f__Cellulomonadaceae;g__;s__	0	1	0	0	0	0	0
Root;k__Bacteria;p__Actinobacteria;c__Actinobacteria;o__Actinomycetales;f__Cellulomonadaceae;g__Cellulomonas;s__	0	0	0	0	0	0	0
Root;k__Bacteria;p__Actinobacteria;c__Actinobacteria;o__Actinomycetales;f__Corynebacteriaceae;g__Corynebacterium;s__	0	36	623	61	0	0	24
Root;k__Bacteria;p__Actinobacteria;c__Actinobacteria;o__Actinomycetales;f__Corynebacteriaceae;g__Corynebacterium;s__aurimucosum	0	0	42	0	0	0	0
Root;k__Bacteria;p__Actinobacteria;c__Actinobacteria;o__Actinomycetales;f__Corynebacteriaceae;g__Corynebacterium;s__kroppenstedtii	0	0	0	0	0	0	0
Root;k__Bacteria;p__Actinobacteria;c__Actinobacteria;o__Actinomycetales;f__Corynebacteriaceae;g__Corynebacterium;s__pilosum	0	0	1	0	0	0	0
Root;k__Bacteria;p__Actinobacteria;c__Actinobacteria;o__Actinomycetales;f__Corynebacteriaceae;g__Corynebacterium;s__stationis	0	0	26	0	0	0	0
Root;k__Bacteria;p__Actinobacteria;c__Actinobacteria;o__Actinomycetales;f__Dermabacteraceae;g__Dermabacter;s__	0	1	0	0	0	0	0
Root;k__Bacteria;p__Actinobacteria;c__Actinobacteria;o__Actinomycetales;f__Microbacteriaceae;g__;s__	0	1	0	0	0	0	0
Root;k__Bacteria;p__Actinobacteria;c__Actinobacteria;o__Actinomycetales;f__Microbacteriaceae;g__CandidatusRhodoluna;s__	0	1	0	1	0	0	1
